# Transcatheter Aortic Valve Implantation in Young Patients: Challenges and Perspectives—A Narrative Review

**DOI:** 10.3390/jcm14196847

**Published:** 2025-09-27

**Authors:** Iulia D. D. Moț, Adela M. Șerban, Alexandru Achim, Ștefan D. C. Moț, Dana Pop

**Affiliations:** 1 4th Department of Internal Medicine, Department of Cardiology Rehabilitation, “Iuliu Hațieganu” University of Medicine and Pharmacy, 400012 Cluj-Napoca, Romania; dorlimot9@gmail.com (I.D.D.M.); pop67dana@gmail.com (D.P.); 2 Cardiology Department, “Niculae Stăncioiu” Heart Institute, 400001 Cluj-Napoca, Romania; adelamserban@yahoo.com (A.M.Ș.); motstefan@gmail.com (Ș.D.C.M.); 3 4th Department of Internal Medicine, Department of Cardiology, “Iuliu Hațieganu” University of Medicine and Pharmacy, 400012 Cluj-Napoca, Romania; 4 Department of Cardiology Rehabilitation, Clinical Rehabilitation Hospital, 400437 Cluj-Napoca, Romania

**Keywords:** TAVI, young patients, bicuspid aortic valves, rheumatic aortic stenosis, outcomes, complications, valve durability, reintervention

## Abstract

Although most commonly diagnosed in the elderly population, aortic stenosis can affect younger patients, being the most frequent valvular disease requiring replacement interventions, either through surgical procedures (SAVR = surgical aortic valve replacement) or through transcatheter aortic valve implantation (TAVI). In young patients, aortic stenosis generally occurs due to congenital malformations, such as bicuspid aortic valves (BAVs), or to rheumatic valve disease, both of which present specific anatomical characteristics. There is an upward trend among young patients regarding TAVI, due to the possibility of avoiding the complications of open-heart surgery while offering a faster recovery, although it is important to note that complications, such as conduction disturbances, paravalvular leaks (PVL), or strokes, can arise. Because of the current lack of long-term data, the implications of these complications among young patients are not well established. Moreover, an important issue among young patients is the durability of the prosthesis, as patient survival is expected to exceed the device’s lifespan. The purpose of this review is to assess the current data on the most common causes of aortic stenosis and outcomes of TAVI in young patients, focusing on subgroups of patients with bicuspid aortic stenosis or rheumatic aortic stenosis, while emphasizing the potential complications, the durability of the aortic prosthesis, and reintervention possibilities.

## 1. Introduction

The development of medical knowledge and technologies in recent decades has led to the emergence of minimally invasive treatment methods, which, in addition to providing clear benefits in terms of early recovery and the avoidance of well-known complications associated with open surgical interventions, are becoming increasingly adopted by patients. An increase in life expectancy within the population has resulted in a higher number of patients being diagnosed with aortic stenosis (AS) [1], and, consequently, in a growing number of aortic valve replacement procedures. Transcatheter aortic valve implantation (TAVI) is a relatively new technique, being a subject of interest in cardiology, interventional cardiology, and cardiovascular surgery, and a continuously evolving minimally invasive procedure. Although the indications for TAVI are well established, there are still certain aspects on which no consensus has been reached, namely, the percutaneous treatment of aortic stenosis in young patients. There are several key aspects that differentiate young patients from the elderly population: the causes of aortic stenosis, the characteristics of younger patients—who are typically at low surgical risk— and the outcomes of complications among them. Considering that young patients usually present fewer comorbidities than older patients, their survival is expected to exceed the device’s lifespan, and thus, certain factors have to be taken into consideration when deciding on their treatment options, including the valve’s durability and future reintervention needs.

The aim of this paper is to review the outcomes of TAVI in young patients, while focusing on the most frequent causes of aortic stenosis in this population group, the complications of TAVI and their implications among young patients, and the problem of the valves’ durability.

## 2. General Considerations: Etiologies of AS in Young Patients

Aortic stenosis, characterized by the obstruction of the left ventricular outflow tract, represents the most frequent valvular pathology requiring valve replacement interventions, with its incidence continuously increasing due to the aging of the population [2,3,4].

The most common cause of aortic valve degeneration is calcific aortic valve disease, followed by congenital pathologies, such as a bicuspid aortic valve (BAV), and rheumatic valvular disease, which is still diagnosed in developing countries [3].

Since the mortality rate of aortic stenosis is very high, at approximately 50% within two years from the onset of symptoms [3,5], and because medical therapy does not halt the natural progression of the disease [2], the only definitive treatment for symptomatic severe aortic stenosis is valve replacement, either through surgical procedures (SAVR = surgical aortic valve replacement) or through transcatheter aortic valve implantation [6].

Although both surgical aortic valve replacement and transcatheter aortic valve implantation have clear indications, case management requires the involvement of the heart team to ensure optimal and individualized treatment for each patient [7].

Considering the new guideline recommendations of the European Society of Cardiology (ESC), the age at which TAVI is indicated is ≥70 years, in patients with symptomatic severe AS whose life expectancy is expected to surpass one year, or asymptomatic severe AS patients with a reduced left ventricular ejection fraction (LVEF) unidentifiable by other causes [7]. According to the American Heart Association (AHA), the indication for transcatheter intervention is an age above 80 years; however, for those between 65 and 80 years, either TAVI or SAVR may be chosen, depending on life expectancy and comorbidities, in patients considered to be at high surgical risk according to the EuroSCORE II and STS-PROM scores [6,8,9]. Furthermore, certain anatomical characteristics and comorbidities may favor one of the valve replacement methods over the other [2].

Even though the initial indication for transcatheter aortic valve implantation was oriented towards inoperable patients, in recent years, indications have expanded to include patients with low surgical risk [10].

Given the age criteria defined by the European and American guidelines, for a better understanding of the implications of TAVI among young patients, we will define this subgroup as being under 75 years of age. Although the prevalence of aortic stenosis increases with advancing age, being over 3.9% in patients older than 70 years, approximately 1.3% of the population under 70 years of age presents with severe aortic stenosis [11].

The main cause of aortic stenosis differs depending on age. Thus, in young patients, aortic stenosis is most often due to congenital malformations, such as bicuspid aortic valves [4], or rheumatic heart disease, as a result of infection with group A beta-hemolytic Streptococcus, while in elderly patients, the main cause is calcific aortic valve disease [3].

A bicuspid aortic valve, a congenital malformation present in 0.5–0.8% of the population, predisposes the affected individual to the development of aortic stenosis, with the average age for surgical valve replacement being approximately 50 years [11]. However, there are also other, much rarer malformations of the aortic valve, such as quadricuspid or unicuspid aortic valves, the latter leading to the clinical presentation of critical aortic stenosis [4]. Typically, the onset of symptoms in patients with congenital malformations of the aortic valve occurs about 20 years earlier compared to in patients with calcific aortic valve disease [12].

The cardiac sequelae of acute rheumatic fever (ARF) constitute rheumatic heart disease and are manifested mainly by changes at the valvular level, such as thickening, fusion, and restriction of the cusps. Compared with calcific aortic valve disease, aortic stenosis caused by ARF is characterized by a lower degree of calcification. In general, the population affected by this pathology is younger, with a mean age of 28 years, and presents multivalvular involvement, with a higher incidence observed in females [13]. Considering that acute rheumatic fever is still diagnosed in developing countries, one of the main problems in patients with severe aortic stenosis caused by ARF is access to transcatheter aortic valve replacement.

## 3. Current Evidence for TAVI in Bicuspid Aortic Valves

Bicuspid aortic valves are divided into three types, according to Hamana et al. [12] based on Sievers et al., depending on the number of raphes present, the positioning of the cusps or raphes, and the functional status of the valve—stenotic, regurgitant, or both [12] (Table 1).

However, the classification proposed by Jilaihawi et al., oriented toward the transcatheter intervention, takes into account the leaflet plane and the level of the commissures, dividing bicuspid valves into three different morphologies [14]:

- Tricommissural, with one commissure fused between two cusps, representing functional or acquired bicuspidia;
- Bicommissural with raphe, with two cusps fused by a fibrous or calcified raphe;
- Bicommissural without raphe, with two fully fused cusps.

The bicuspid aortic valve is characterized by anatomical complexity, as these valves have a larger size compared to tricuspid valves, contain more calcium, and are associated with weakness of the aortic wall, while patients more frequently present with aortic insufficiency and ascending aortic ectasia (AAE) [15].

Extensive calcifications of the raphe and cusps are associated with higher two-year mortality, aortic annular injury, and paravalvular regurgitation after TAVI, compared to patients without these morphological features. An observational retrospective study on 1034 BAV patients, with a mean age of 74.7, who underwent TAVI with balloon-expandable valves (BEVs) in 71.6% of cases was performed by Yoon et al. [16]. The results indicate that 2-year all-cause mortality was higher among type 1 BAV patients with excess raphe calcifications than among type 1 noncalcified BAV or type 0 BAV patients (17.7% vs. 9.3% vs. 2.4%; log-rank *p* = 0.001). In patients with excess raphe and cusp calcification, aortic root injury was more frequently observed than in those with only one or none of these anatomical components (4.5% vs. 0.7% vs. 0.9%; *p* < 0.001). Paravalvular regurgitation of more than moderate grade had a higher incidence among these patients (6.5% vs. 2.5% vs. 1.6%; *p* = 0.002). Thus, the extent of calcifications and characterization of the bicuspid aortic valve could determine the post-TAVI prognosis [16].

Morphological changes occurring at the level of the ascending aorta, such as ectasia or even aneurysm, together with the patient’s history (genetic, personal—aortic dissection, other concomitant valvulopathies, or an increase in the diameter of the aorta), indicate the need for intervention both on the valve and on the ascending aorta [17]. However, correction of the hemodynamic parameters at the aortic valve after TAVI could decrease the incidence of ascending aortic dilatation, and thus the need for intervention on it [17].

However, according to Fan et al. [18], performing TAVI on bicuspid aortic valves associated with ascending aortic ectasia could result in higher long-term mortality. A retrospective study conducted on a group of 111 patients with a mean age of 74.63 ± 7.1 with bicuspid aortic valves and aortic stenosis treated with TAVI with self-expanding valves (SEVs), comparing patients with AAE (diameter over 40 mm) to patients without ascending aortic dilatation, found higher mortality among patients with AAE (26.5% vs. 11.2%, *p* = 0.022). Deceased patients presented more pronounced progression of aortic ectasia (*p* = 0.001) and a lower reduction in ascending aorta diameter (7.1% vs. 53.7%, *p* = 0.001). Predictors of death beyond four years after TAVI were age, ascending aorta dilatation, and postoperative left ventricular systolic pressure (*p* = 0.034, *p* = 0.038, *p* = 0.044). Thus, when tolerated, SAVR with ascending aorta replacement could be the preferred treatment method in these patients [18]. Therefore, a bicuspid aortic valve is not a contraindication for TAVI, but requires attention to these parameters [15].

In a study conducted by Makkar et al. [19]. on 3168 pairs of patients matched by propensity score, with a mean age of 69 years, when comparing patients at low surgical risk treated with TAVI with balloon-expandable valves, no differences were observed in terms of mortality between patients with BAVs versus tricuspid aortic valves at 30 days (0.9% vs. 0.8%; HR, 1.18 [95% CI, 0.68 to 2.03]; *p*  =  0.55) and 1 year after the intervention (4.6% vs. 6.6%; HR, 0.75 [95% CI, 0.55 to 1.02]; *p*  =  0.06). The incidence of stroke at 30 days (1.4% vs. 1.2%; HR, 1.14 [95% CI, 0.73 to 1.78]; *p*  =  0.55) and 1 year (2.0% vs. 2.1%; HR, 1.03 [95% CI, 0.69 to 1.53]; *p*  =  0.89) did not differ between the two population groups. Furthermore, echocardiographic parameters such as the valve area, transvalvular gradient, and moderate-to-severe paravalvular regurgitation were similar between the two groups at the same time intervals. The incidence of procedural complications, such as conversion to surgical intervention (0.4% vs. 0.4%, *p* = 0.85), coronary artery obstruction (CO) (0.2% vs. 0.1%, *p* = 0.34), and permanent pacemaker implantation (PPM) (6.2% vs. 5.2%, *p* = 0.07), was also similar between the BAV and tricuspid valve patients [19].

The randomized clinical trial NOTION-2 compared the outcomes of surgical aortic valve replacement versus transfemoral TAVI in 370 young patients (with a mean age of 71.1 years) with symptomatic severe aortic stenosis and low surgical risk, including 100 patients with BAVs, all eligible for both interventions. Exclusion criteria were complex and composed of severe comorbidities, hemodynamic instability, and anatomical particularities, such as BAVs with AAE. Overall, similar results were demonstrated for both types of intervention regarding the risk of all-cause death, stroke, and rehospitalization—10.2% in the TAVI-treated group vs. 7.1% in the surgical cohort (HR, 1.4, 95% CI 0.7–2.9; *p* = 0.3). Patients with bicuspid aortic valves were found to have a higher risk of death, stroke, and rehospitalization after TAVI than after surgery at 1 year after the intervention (14.3% vs. 3.9%, HR, 3.8, 95% CI 0.8–18.5; *p* = 0.07). Regarding stroke, partial or no resolution of neurological deficit was encountered in two bicuspid AS patients after TAVI; these were cases where a cerebral embolic protection device was not used during the intervention. More-than-moderate paravalvular regurgitation after TAVI in patients with bicuspid aortic valves was also more frequently observed (9.1%) compared to in patients with tricuspid aortic valves (3.1%). Regarding SAVR in bicuspid AS patients, the primary event rate was lower as compared to in tricuspid AS patients (3.9% compared to 8.3%), which could be partially explained by the younger age and anatomical features associated with BAVs. On the other hand, there were no significant differences in the incidence of pacemaker implantation (14.6% vs. 15.2%) between patients with bicuspid and tricuspid aortic valves treated with TAVI [20].

Nevertheless, according to a recent retrospective study conducted by Li et al. [21] on 2553 patients, including 439 with BAVs, patients with this malformation, especially type 0 BAV, who were treated with TAVI had better long-term outcomes compared to patients with tricuspid aortic valves in regard to all-cause (11.0% [5.3–16.3%] and 34.5% [27.8–40.7%] versus 45.5% [43.0–48.0%]; log-rank *p* < 0.0001) and cardiovascular mortality (4.3% [0.5–8.0%] and 18.1% [12.4–23.5%] versus 27.9% [25.4–30.3%]; log-rank *p* < 0.0001). Lower all-cause (23.6% [17.9–28.8%] versus 41.7% [28.5–52.5%]; aHR: 1.63 [95% CI: 1.05 2.51]; *p* = 0.028) and cardiovascular mortality (11.0% [6.6% 15.2%] versus 24.4% [12.4–34.9%]; aHR: 2.01 [95% CI: 1.06–3.79]; *p* = 0.032) was also found among BAV patients treated with self-expanding valves than among those treated with BEVs. Multiple factors were associated with an increased risk of mortality, such as advanced age, high surgical risk, different comorbidities (hypertension, diabetes, coronary artery disease, cerebrovascular disease, chronic kidney disease, atrial fibrillation), and a prior permanent pacemaker. The type 0 BAV patients were younger, and had the lowest incidence of permanent pacemaker implantation rates (14.0% as compared to 21.6% in type 1 BAV patients and 14.7% in tricuspid valve patients, *p* = 0.011), and this could be explained by the morphology of the valve, which forces the placement of the prosthesis above the aortic annulus, and thus away from the conduction system. The risk of more-than-moderate paravalvular leak (PVL) was highest among these patients (6.0% versus 4.6% in type 1 BAV and 2.5% in tricuspid valves, *p* = 0.011); however, because of their young age and possibly stronger cardiac anatomy, the patients’ prognosis could exceed the risk of paravalvular leak [21].

The differences in outcomes between type 0 and type 1 bicuspid aortic valves are hypothesized to occur because of the degenerative processes that are present on top of the congenital malformation in type 1 BAV. Considering the fact that previous studies have not stratified bicuspid aortic valve patients into subgroups according to morphology, this could be the reason why previous findings suggest similarity in the outcomes of BAV and tricuspid aortic valve patients treated with TAVI [21].

To ensure an optimal transcatheter intervention in patients with bicuspid aortic valves and severe aortic stenosis, particular attention must be paid to anatomical parameters, as well as to possible future reintervention needs, such as re-replacement of the aortic prosthesis due to its degeneration, coronary access (CA) in the context of coronary angiography, and the necessity of permanent pacemaker implantation [19].

Clinical practice: Considering the information presented above, as BAVs are associated with a higher degree of calcifications and with ascending aortic ectasia, such patients could present an increased risk of mortality, and thus, patients should be carefully selected when deciding on the optimal intervention method. This concept is further strengthened by the findings of Li et al., which underline the importance of BAV characterization [21]. However, when comparing SAVR with TAVI in BAV patients, a higher risk of mortality was found among the transcatheter-treated patients [20]. The 2025 European Society of Cardiology Guidelines on valvular heart disease conclude that SAVR remains the primary intervention, especially in younger patients, but TAVI may be chosen in patients with increased surgical risk [7]. Still, there are some limitations regarding BAV patients treated with TAVI: there was a rather small number of patients with BAVs included in the NOTION-2 trial—the only randomized clinical trial that studied the outcomes of TAVI among patients with this malformation; furthermore, valve subtype characterization was not performed, and the prosthesis choice was not defined [20]. Therefore, more research focused on BAV patients treated with TAVI is needed, as the incidence of BAVs is high, especially among younger patients.

## 4. Rheumatic Valve Disease and TAVI—An Ongoing Question

So far, patients with rheumatic aortic stenosis have been excluded from major trials due to the morphological particularities of the aortic valve, with only small studies currently available, and no consensus regarding the use of a specific type of prosthesis [13].

Rheumatic valve disease is characterized by fibrosis and commissural fusion, which create an elastic band at the level of the aortic valve that predisposes the affected individual to embolization of the aortic prosthesis, and therefore, balloon predilatation may be necessary before implantation [22]. The reduced degree of calcification complicates the transcatheter intervention by creating difficulties in anchoring the aortic prosthesis and predisposes the individual to annular rupture, prosthesis embolization, paravalvular regurgitation, and conduction disorders [13,22].

In a prospective cohort study conducted by Elkaialy et al. [22], the outcomes of TAVI in 54 patients, with a mean age of 72.35 ± 5.86, diagnosed with AS secondary to ARF were compared to those in propensity score-matched 54 patients with severe calcific aortic stenosis, at low surgical risk, through periodic follow-up at 30 days and 6 months postoperatively. Preoperatively, patients with rheumatic aortic stenosis presented different morphological features (lower transvalvular gradients—45.63  ±  11.86 mmHg versus 49.91  ±  12.43 mmHg, smaller annular diameters—22.36  ±  2.31 versus 23.53  ±  2.3, and a lower degree of calcification) and ARF mitral valve involvement, in conformation to the primary pathology. During the procedure, certain aspects differed between the two patient groups, such as the performance of predilatation and the depth of implantation, which were significantly higher in the rheumatic cohort, and a self-expanding prosthesis was most frequently used in both patient cohorts (rheumatic AS 79.63% and calcific AS 77.78%). Postoperatively, no significant differences were found regarding mortality, rhythm disorders (*p* = 0.363), conduction disorders (*p* = 0.165), or the rate of mild paravalvular regurgitation (*p* = 0.102) between the two patient groups. After TAVI, overall, the most frequent conduction disorders detected were transient, such as transient left bundle branch block (LBBB) (20.4%) and transient atrio-ventricular block (9.3%). After a 6 month follow-up period, all cause death was 3.7% in the rheumatic AS cohort [22].

In the study conducted by Mentias et al., comparisons were made between SAVR and TAVI in 1159 patients diagnosed with rheumatic AS, as well as TAVI in 88,554 patients with severe non-rheumatic AS (including bicuspid aortic valves), with a mean age of 79.4 ± 8.1 years [23]. The patients were divided into three groups, as shown in the following Table 2.

After propensity score adjustment, no significant differences were found between patients with rheumatic aortic stenosis treated by SAVR or TAVI regarding in-hospital and 30-day mortality (3.5% versus 2.4%, *p* = 0.6; and 3.2% versus 3.6%, *p* = 0.9), and the incidence of stroke at 30 days after the intervention (2.8% versus 2.4%, *p* = 0.8). After 1 year, similar results were found of no statistical difference regarding mortality between the two groups, with a rate of 8.9% for SAVR and 13.1% for TAVI, *p* = 0.2. Patients treated surgically more frequently presented in-hospital complications, such as acute renal failure (22.3% versus 11.9%, *p* = 0.02), newly diagnosed atrial fibrillation (21.1% versus 2.2%, *p* < 0.001), and cardiogenic shock (5.7% versus 1.5%, *p* = 0.047), compared with patients with rheumatic aortic stenosis treated by TAVI. In addition, surgical aortic valve replacement increased the risk of right ventricular dysfunction, while patients with rheumatic heart disease frequently present right ventricular dysfunction [23].

The minimally invasive treatment groups (rheumatic vs. non-rheumatic AS) showed similar results in terms of in-hospital mortality (2.2% vs. 2.6%, *p* = 0.6), 30-day mortality (3.6% vs. 3.7%, *p* = 0.95), 30-day incidence of stroke (2.0% versus 3.3%, *p* = 0.1), and the need for a new permanent pacemaker (12.2% versus 11.4%, *p* = 0.7), but 242 (0.3%) patients with non-rheumatic aortic stenosis required reintervention on the aortic valve—124 redo-TAVI and 118 SAVR [23]. Although this number is not high, the finding is particularly important considering that acute rheumatic fever is diagnosed in developing countries, where access to modern therapies is limited.

Even though the short-term outcomes of TAVI in patients with rheumatic aortic stenosis appear to be similar to those in patients with calcific aortic stenosis, as well as there being positive aspects in terms of a low incidence of in-hospital complications and reintervention rates according to the presented data above, randomized clinical trials with longer follow-up periods are needed, especially in younger patients, who are expected to present better survival than older patients.

Clinical practice: Similarly to in BAV patients, the use of TAVI in rheumatic aortic stenosis patients requires considerable awareness of anatomical parameters and specific procedural details, but because of the underpowered sample sizes of the abovementioned studies, in order to achieve the best standards in clinical practice, it would be suitable to await more results. A potential practical limitation among rheumatic AS patients could be the multivalvular involvement of this disease [13], and this aspect should be taken into consideration when deciding on treatment strategies.

## 5. Young Patients—A Shift Towards TAVI

The indications for transcatheter aortic valve implantation have expanded in recent years to include patients with low surgical risk. Following the PARTNER-3 and Evolut Low-Risk trials, a lower risk of death, stroke, and rehospitalization was demonstrated after the intervention compared with after open-heart surgery. However, low surgical risk is not always equivalent to the patient’s age, even though low-risk patients are generally younger [15,24].

According to a single-center retrospective study conducted by Ancona et al. [25] on 129 patients with a mean age of 63.6 ± 6 and at high surgical risk (overall STS-mortality score of 7.35%), including patients with BAVs, treated with first- and second-generation TAVI prostheses, a device success rate of 89% was observed, with low rates of stroke (2.3%) and in-hospital mortality (6.2%). Moreover, a low rate of permanent pacemaker implantation need (5.4%) and low incidence of reintervention (3.9%) was also found among these patients. The cohort treated with second-generation valves received self-expanding valves in more than half of the cases, and among them, a significantly higher rate of left bundle branch block was diagnosed compared to with first-generation valves (16.9% versus 5.2%, *p* = 0.039). In this study, the three-year all-cause mortality was 34%, with no difference between the first- and second-generation valves, and probably attributable to comorbidities, with the most important predictors being high surgical risk (HR, 3.81, multivariate 1.06–13.7; *p* = 0.040), diabetes mellitus (HR, 4.69 multivariate 1.72–12.8; *p* = 0.003), and chronic kidney disease (HR, 2.91, multivariate 1.08–7.85; *p* = 0.035) [25].

Bække et al. [26] conducted a retrospective multicenter study between 2010 and 2019 comparing three-year mortality in 459 young patients, with a mean age of 61 years, treated with TAVI—mainly because of surgical contraindications—with a control group of 1836 patients with similar demographic characteristics (age, sex) and comorbidities, but without aortic stenosis, and who were thus not treated. The results of this study showed a significantly higher all-cause mortality in the TAVI group compared to the population matched for age, gender, and comorbidity (33.1% vs. 7.5%, HR, 6.5, 95% CI 4.5–9.6, *p* < 0.001), especially in those with chronic diseases—heart failure (HR, 1.9; 1.1–3.1), chronic obstructive pulmonary disease (HR, 2.4; 1.4–3.9), chronic kidney disease on dialysis (HR, 1.8; 0.9–3.9)—as well as in those with inactive conditions, such as cancer or a history of cardiac surgery or stroke (HR, 6.8–24.7). The highest 3-year mortality was observed in TAVI recipients on chronic dialysis compared to the matched population (50.3% vs. 22.1%). However, it is important to note that the control group was homogeneous, with all participants being from the same country, and matching was performed for a single comorbidity, whereas most patients in the TAVI-treated group had multiple comorbidities. The rates of the post-procedural complications identified, such as permanent pacemaker implantation (12%), reintervention (3%), vascular complications (6%), stroke (2%), and acute kidney injury (8%), were low. Thus, even though the mean STS-PROM score would be considered low (2.6% in this study), young patients with multiple comorbidities should be considered high-risk [26].

Post-TAVI outcomes in patients under 60 years of age demonstrate a low complication rate and similar in-hospital mortality to that observed after SAVR. According to a retrospective observational study conducted by Gad et al. [27] on 3672 TAVI patients aged between 18 and 59, matched to SAVR-receiving patients, the in-hospital mortality rate was similar (2.9% vs. 3.0%, *p* = 0.77). The incidence of in-hospital complications, such as cardiogenic shock (5.5% vs. 8.8%, *p* < 0.001), aortic dissection (3.2% vs. 9.3%, *p* < 0.001), cerebrovascular events (1.9% vs. 3.3%, *p* < 0.001), and acute kidney injury (13.0% vs. 21.3%, *p* < 0.001), was lower post-TAVI, and no significant differences were detected between the interventions regarding the need for permanent pacemaker implantation (5.2% vs. 4.9%, *p* = 0.59), even though patients treated with TAVI had multiple preoperative comorbidities. Furthermore, during the last years, a decline in in-hospital mortality and the 30-day readmission rate after TAVI was observed [27].

A study led by Nelson et al. [28] from 2013 to 2018 on adults aged 18–55 from the Congenital Heart Surgery Database and Adult Cardiac Surgery Database compared the outcomes of SAVR and TAVI, including in patients with congenital heart disease. The results showed lower, but not statistically different, 30-day mortality (1.6% vs. 2.9%, *p* = 0.076) and morbidity rates (16% vs. 17%, *p* = 0.6), and lower rates of stroke (0.9% vs. 2.4%, *p* = 0.002), after isolated SAVR than after TAVI. A higher rate of morbidity after TAVI was found in patients with congenital heart disease. Even though the most frequently used valves were bioprosthetic, in the last few years an increase by 167% was observed for TAVI [28].

According to the DEDICATE trial, TAVI was found to be non-inferior to SAVR regarding mortality and stroke (fatal and non-fatal) at 1 year after the intervention among patients at low-to-moderate surgical risk (5.4% vs. 10.0%, HR: 0.53, 95% CI 0.35–0.79, *p* < 0.001). This prospective, multicenter, randomized controlled trial consisted of 1414 patients eligible both for TAVI and SAVR, with a mean age of 74 ± 4 years, while patients with severe comorbidities or a life expectancy under 1 year, as well as patients with variant anatomical features—BAV or uncalcified aortic stenosis—were excluded. BEVs were used in more than half of the TAVI-treated group (61.4%). In the SAVR group, approximately 78% of patients received a stented bioprosthesis. At one year, death from any cause appeared in 2.6% of patients in the TAVI group versus 6.2% in the SAVR group (HR, 0.43, 95% CI 0.24–0.73). The incidence of conduction disturbances requiring permanent pacemaker implantation (11.8% vs. 6.7%, HR, 1.81; 95% CI 1.27–2.61), prosthetic valvular dysfunction (1.6% vs. 0.6% HR, 2.44; 95% CI 0.87–8.15), aortic valve reintervention (0.6% vs. 0.3%, HR, 1.70, 95% CI 0.38–9.78), and vascular complications (7.9% vs. 0.7%, HR, 10.64; 95% CI 4.84–28.94) appeared to be higher among patients treated with TAVI, while stroke (4.7% vs. 2.9%, HR, 0.61, 95% CI 0.35–1.06), newly diagnosed atrial fibrillation (30.8% vs. 12.4%, HR, 0.36; 95% CI 0.28–0.46), and major bleeding (17.2% vs. 4.3%, HR, 0.24; 95% CI 0.16–0.35) were more frequent in the surgical cohort [29,30].

The one-year results of the NOTION-2 trial regarding tricuspid AS patients showed similarities between the two intervention methods regarding all-cause mortality, stroke, and rehospitalization—8.7% in the TAVI group vs. 8.3% in the surgery cohort (HR, 1.0, 95% CI 0.5–2.3; *p* = 0.9). The rates of death from any cause appeared in 1.1% of TAVI and 2.1% of SAVR patients (HR, 2.0, 95% CI 0.4–10.7, *p* = 0.4) [20].

The outcomes of the secondary endpoints in the overall TAVI cohort (including bicuspid and tricuspid AS patients) showed similarities between the two interventions: major vascular complications (1.6% vs. 1.6%, HR, 1.0, 95% CI 0.2–4.8, *p* = 1.0) and myocardial infarction (MI) (2.1% vs. 1.6%, HR, 1.3, 95% CI 0.3–5.9; *p* = 0.7). Lower risks of major bleeding (4.8% vs. 17.5%, HR, 0.3, 95% CI 0.1–0.5; *p* < 0.001), newly diagnosed atrial fibrillation (3.2% vs. 41.7%, HR, 0.06, 95% CI 0.03–0.2; *p* < 0.001), and severe patient–prosthesis mismatch (10.1% vs. 19.4%, HR, 0.5, 95% CI 0.3–0.9, *p* = 0.02) were observed in the TAVI cohort. A total of 72.7% of the TAVI patients were treated with a self-expanding prosthesis, and a cerebral embolic protection device was used in 15.5% of the TAVI-treated patients. A higher risk of stroke (5.4% vs. 1.6%, HR, 3.3, 95% CI 0.9–12.0, *p* = 0.05), conduction disturbances requiring pacemaker implantation (15.1% vs. 8.0%, HR, 2.0; 95% CI 1.1–3.8; *p* = 0.03), and more-than-moderate paravalvular regurgitation (4.7% vs. 0%; *p* = 0.005) was observed in the TAVI cohort compared with those treated by SAVR, although it is important to note that the inclusion criteria also encompassed patients with bicuspid aortic valves. At present, results are limited to one year after intervention, and these show similar rates of aortic reintervention after TAVI and SAVR (1.1% vs. 2.2%, HR, 0.5, 95% CI 0.1–2.7, *p* = 0.4) [20].

In the PARTNER-3 multicenter, randomized clinical trial, one-year postoperative outcomes were compared between transfemoral TAVI with third-generation balloon-expandable valves and SAVR in 950 patients with severe symptomatic AS or asymptomatic AS with a reduced ejection fraction (<50%), for which both interventions are suitable, who had a mean age of 73 years, low surgical risk (mean STS-PROM score of 1.9%), and no major anatomical or clinical particularities. The exclusion criteria were complex and included patients with anomalies of the aortic valve, cardiovascular comorbidities, severe comorbidities, hemodynamic instability, and a life expectancy under 2 years [31].

TAVI was performed in 496 patients and was shown to be superior regarding the risk of death, stroke, and rehospitalization, both at 30 days (4.2% vs. 9.3%, HR, 0.45, 95% CI 0.27–0.76;) and at 1 year (8.5% vs. 15.1% HR, 0.54; 95% CI, 0.37–0.79, *p* = 0.001). At 1 year, the individual rates of death from any cause (1.0% vs. 2.5%, HR, 0.41, 95% CI 0.14–1.17) and stroke (1.2% vs. 3.1%, HR, 0.38, 95% CI 0.15–1.00) were lower for the TAVI-treated cohort. The TAVI-treated group also had shorter hospital stays (3 vs. 7 days, *p* < 0.001), a lower risk of poor post-procedural outcomes (3.9% vs. 30.6%, *p* < 0.001), a faster improvement in symptoms at 30 days according to the NYHA (New York Heart Association = NYHA) functional class—NYHA class II-IV (19.7% for TAVI vs. 33.3% SAVR), and an improvement from baseline in the 6 min walk test (32.0% vs. 7.4%) and the Kansas City Cardiomyopathy Questionnaire (KCCQ) (37.8% vs. 12.8%). However, at one year, a major difference was only observed in the 6 min walk test, with 32.2% of TAVI patients having an improvement from baseline, versus 17.1% of SAVR patients. At 30 days, the incidence of stroke (0.6% vs. 2.4%, HR, 0.25, 95% CI 0.07–0.88, *p* = 0.02) and newly diagnosed atrial fibrillation (5.0% vs. 39.5%; HR, 0.10; 95% CI, 0.06–0.16; *p* < 0.001) was lower in TAVI-treated patients, and remained lower at one year (stroke—1.2% vs. 3.1%, HR, 0.38, 95% CI 0.15–1.00; atrial fibrillation—7.0% vs. 40.9%, HR, 0.13, 95% CI 0.09–0.20). The incidence of major vascular complications (2.8% vs. 1.5%, HR, 1.83, 95% CI 0.74–4.55), permanent pacemaker implantation (7.3% vs. 5.4%, HR, 1.39, 95% CI 0.83–2.33), moderate-to-severe paravalvular regurgitation (0.6% vs. 0.5%, 0.1%, 95% CI −0.91–1.15%), and coronary obstruction (0.2% vs. 0.7%, HR, 0.30, 95% CI 0.03–2.93) was similar between the two patient groups at one year. However, at one year post-TAVI, patients more frequently developed left bundle branch block (23.7% vs. 8.0%, HR, 3.43; 95% CI, 2.32 to 5.08) and mild paravalvular leak [31].

At 5 years after the intervention, results from the PARTNER-3 trial regarding death, stroke, and rehospitalization demonstrated similarity between the two interventions, with primary endpoints occurring in 22.8% of patients in the TAVI cohort versus 27.2% in the SAVR cohort (HR, 0.79, 95% CI 0.61–1.02, *p* = 0.07). Individually, death from any cause and stroke at 5 years in the TAVI and SAVR groups, respectively, were similar (death from any cause 10.0% vs. 8.2%, HR, 1.23, 95% CI 0.79–1.90; stroke 5.8% vs. 6.4%, HR, 0.87, 95% CI 0.51–1.48). A persistently lower incidence of atrial fibrillation (13.7% vs. 42.4%, HR, 0.25, 95% CI 0.19–0.34) and major bleeding (10.2% vs. 14.8%, HR, 0.65, 95% CI 0.45–0.95), but a higher incidence of more-than-mild paravalvular regurgitation (20.8% vs. 3.2%), valve thrombosis (2.5% vs. 0.2%, HR, 10.52, 95% CI 1.37–80.93), and pacemaker implantation (13.5% vs. 10.4%, HR, 1.33, 95% CI 0.90–1.96), was observed in the minimally invasive-treated group. The rates of aortic valve reintervention were also similar at 5 years, with 2.6% in the TAVI-treated group and 3.0% in SAVR patients (HR, 0.86, 95% CI 0.39–1.92). Mild paravalvular regurgitation did not significantly affect 5-year mortality, which reached 9.1% in patients with no PVL at 30 days post-procedure, versus 11.1% in those with mild PVL 30 days post-TAVI (HR, 0.78, 95% CI, 0.42–1.45) [32].

The Evolut Low-Risk multicenter, prospective, randomized clinical trial, which compared the 5-year outcomes of 730 TAVI patients treated with self-expanding valves to those of 684 SAVR patients at low surgical risk (STS-PROM scores of 2.0% ± 0.7% for TAVI, and 1.9% ± 0.7% in the SAVR group, respectively), the mean age being 74 years [33]. The exclusion criteria consisted of anatomical particularities, severe comorbidities, hemodynamic instability, pregnancy, and a life expectancy under 2 years [34]. The 5-year outcomes demonstrated similarity between the two interventions regarding all-cause mortality or disabling stroke (15.5% in the TAVI cohort versus 16.4% in SAVR, HR, 0.90, 95% CI 0.69–1.18; *p* = 0.47). All-cause mortality occurred in 13.5% of the TAVI patients and 14.9% of the SAVR patients, respectively (HR, 0.88, 95% CI 0.66–1.17, *p* = 0.39), while stroke was diagnosed among 9.5% of TAVI patients and 8.6% of SAVR patients (HR, 1.11, 95% CI 0.77–1.59, *p* = 0.58) [33].

When comparing outcomes at the prosthesis level, at 5 years post-TAVI, patients presented superior valve hemodynamics compared with those with surgically implanted valves, with lower mean gradients (10.7 ± 6.6 mmHg vs. 12.8 ± 6.9 mmHg; *p* < 0.001) and larger effective orifice areas (EOAs) (2.1 ± 0.6 cm^2^ vs. 1.9 ± 0.6 cm^2^; *p* < 0.001), but higher rates of more-than-mild paravalvular leak (14.7% vs. 0.5%, Risk Difference 14.1, 95% CI 10.8–17.5; *p* < 0.001). The need for reintervention on the valve (3.3% vs. 2.5%, HR, 1.30, 95% CI 0.66–2.56; *p* = 0.44), as well as the incidence of infective endocarditis (1.4% vs. 2.5%, HR, 0.52, 95% CI 0.23–1.20, *p* = 0.12) or valve thrombosis (0.9% vs. 0.6%, HR, 1.36, 95% CI 0.38–4.82, *p* = 0.63), was similar between the TAVI and SAVR groups. Although TAVI-treated patients initially showed earlier improvement in quality of life compared with the surgical group (KCCQ of 88.6 vs. 78.7), these results equalized in the first year post-procedure (KCCQ 90.6 vs. 90.5). A lower risk of newly diagnosed atrial fibrillation was noticed in the TAVI cohort (16.3% vs. 41.2%, HR, 0.32, 95% CI 0.25–0.39, *p* < 0.001). However, the minimally invasive-treated group presented higher rates of permanent pacemaker implantation (27.0% vs. 11.3%, HR, 2.70, 95% CI 2.04–3.55, *p* < 0.001), with a higher mortality at 5 years among these patients compared to among non-receiving pacemaker patients (16.6%, 95% CI: 10.9–24.9% vs. 12.1%, 95% CI: 9.5–15.2%) [33].

The following tables provide a synthesis of the abovementioned studies, with a focus on the primary endpoints and most important complications (Table 3 and Table 4).

Clinical practice: Although the results from the above mentioned studies are favorable to TAVI, with it showing similar or lower rates of mortality compared to SAVR, it is important to remember the fact that exclusion criteria were strict and real-life patients are usually more complex, and this could be a potential limitation of the current evidence. Even though some patients could be perceived as being at low surgical risk, they can present severe comorbidities that increase the risk of death after the procedures. Moreover, younger patients are more likely to present with complications of congenital heart disease, where higher morbidity after TAVI could be expected. There are certain aspects that are of major interest among younger patients—such as stroke, conduction disturbances that require permanent pacemaker implantation, coronary obstruction, the long-term durability of the valve, and reintervention strategies—with the idea being to offer these patients a minimal impact of these complications. However, for a more comprehensive understanding and to confirm the impact of complications, long-term follow-up of these patients is required.

## 6. Complications of TAVI in Younger Patients

According to a retrospective cohort study conducted by Harvey et al., in the United States, the number of aortic valve replacement procedures has increased in recent years, with a reduction in the number of post-procedural complications, the decrease being more pronounced after TAVI [35]. “The Big 5” is a term used to describe five of the most common complications of transcatheter aortic valve implantation, all of which are associated to some extent with postoperative mortality. These are conduction disturbances requiring permanent pacemaker implantation, disabling stroke, acute kidney injury, vascular complications and major bleeding, and moderate-to-severe paravalvular leak [36]. Coronary obstruction, although rare, is a complication associated with high mortality [24].

During the procedure, the most commonly occurring are vascular complications, regardless of increases in operator experience and constant improvement of materials [37]. According to the VARC-3 criteria, there are multiple vascular and access site complications, divided into major and minor outcomes. Major complications refer to complications of the aorta (dissection or rupture), arterial or venous injury (pseudoaneurysm, hematoma, dissection, rupture, thrombosis, infection, etc.), embolization from a vascular source, imminent endovascular or surgical intervention, or closure device failure resulting in death, serious bleeding, ischemia, amputation, or permanent neurological deficit. Minor complications are composed of the same criteria, but do not result in the above mentioned outcomes [38]. A minimalist approach, with a classical transfemoral access and a distal radial artery secondary access, could decrease the incidence of these complications while improving the operator’s ergonomics [37]. This finding is further sustained by Versteeg et al.’s randomized controlled trial, where an upper-extremity secondary access was found to lead to a lower risk of access site bleeding, but at the cost of a longer duration of the intervention and more frequent access failure [39].

The most common periprocedural complications in patients treated with TAVI are conduction disturbances [40]. The most frequently recorded conduction disorder in post-TAVI patients is left bundle branch block, while the most severe is high-grade atrio-ventricular block, both of which occur due to the proximity of the conduction system to the valve implantation site. Certain factors predispose an individual to an increased risk of requiring permanent pacing after TAVI, namely, male sex, advanced age, pre-existing conduction disorders (right bundle branch block—according to some studies the most common predictor; left anterior fascicular block), periprocedural atrio-ventricular block, the presence of calcifications in the left ventricular outflow tract, and the type of prosthesis used [41].

Implantation of a permanent pacemaker is a procedure associated with certain risks (cardiac device infections, lead dislodgement, pneumothorax, pericardial effusion, etc.), and may predispose an individual to the development of pacemaker-induced cardiomyopathy (PICM). This cardiomyopathy is defined as a reduction in the LVEF of ≥10%, resulting in an ejection fraction of <50% (in the absence of another identifiable cause), with a pacing burden of >20%, and it is associated with increased mortality and poorer postoperative outcomes [24,42].

A retrospective study was conducted by Li et al. [42] on 325 young patients (aged 18–59) with permanent pacemakers implanted between 1986 and 2015, with 46.3% of patients younger than 50 at the time of the intervention. The results of the study showed that 56% of patients had a high pacing burden at follow-up, and were more likely to present with a reduced ejection fraction and pre-implant rhythm or conduction disorders. After a median follow-up of 11.5 years, PICM was diagnosed in 11.7% of patients (median time to PICM of 5.2 years), and these patients presented with a very high right ventricular pacing burden compared to patients without PICM (100% vs. 13.5%, *p* < 0.001). Characteristic differences between PICM and No PICM patients were an age over 50 years (HR, 2.4, 95% CI 1.2–5.0, *p* = 0.013), a reduced left ventricular ejection fraction (HR, 2.5, 95% CI 1.1–5.4, *p* = 0.022), and prior atrio-ventricular block (HR, 2.7, 95% CI 1.3–5.6, *p* = 0.007). Patients aged between 18 and 49 years, especially without prior atrio-ventricular block, were less likely to develop PICM; however, the time to diagnosis of PICM ranged between 2.5 months and 16.9 years in this study, and thus, in patients with a high pacing burden, the onset of this cardiomyopathy could be delayed, and as such, a prolonged and high pacing burden could be considered a risk factor among young patients. This study further emphasizes the importance of identifying risk factors for a high right ventricular pacing burden in addition to PICM risk factors to help predict the incidence of this cardiomyopathy [42].

Moreover, a single-center retrospective investigation led by Cho et al. [43] on 1418 patients—without previously diagnosed heart failure and with a preserved ejection fraction—who underwent PPM implantation between 1994 and 2015. Patients with biventricular pacemakers, single-chamber atrial pacemakers or cardioverter-defibrillators, and other-cause cardiomyopathies were excluded, and the remaining number of patients was 618. A total of 14.1% of the patients developed PICM during a median time of 3.5 years. Compared to non-PICM patients, the PICM cohort presented with a lower baseline ejection fraction (61  ±  5 vs. 63  ±  6, *p* = 0.001), a larger baseline QRS complex (121  ±  33 ms vs. 109  ±  33 ms, *p* = 0.001), a higher likelihood of having had prior left bundle branch block (11.5% vs. 1.9%, *p* < 0.001), a larger paced QRS duration (154  ±  37 vs. 130  ±  40, *p* < 0.001), broader corrected QT intervals (467  ±  53 vs. 441  ±  66, *p* < 0.001), and a higher right ventricular pacing burden (84  ±  29% vs. 65  ±  38%, *p* < 0.001). Following multivariate analysis, independent predictors of PICM included previous LBBB (OR, 4.22, 95% CI 1.34–13.3, *p* = 0.01), an increase in the paced QRS duration (OR, 1.11, 95% CI 1.01–1.21, *p* = 0.03), and a high ventricular pacing frequency (OR, 1.01, 95% CI 1.00–1.02, *p* = 0.02). After a median follow-up of 7.2 years, patients with PICM had a higher risk of all-cause death or heart failure admission (54.0% vs. 38.3%, adjusted HR, 2.93, 95% CI 1.82–4.72, *p* < 0.001), cardiac death (42.7% vs. 1.6%, adjusted HR, 5.70, 95% CI 1.19–21.8, *p* = 0.01), and heart failure admission (47.2% vs. 7.7%, adjusted HR, 5.11, 95% CI 2.79–9.38, *p* < 0.001) [43].

Newlon et al. [44] conducted a retrospective study on a small group of 29 pediatric patients treated with TAVI with balloon-expandable valves; the incidence of third-degree atrio-ventricular block and PPM implantation need was 3.6%. In line with the results of Li et al. [42], patients who developed conduction disturbances post-TAVI were older (17.4 years vs. 14.4 years, *p* = 0.015); however, no other baseline differences were noticed between patients with and without conduction disturbances. Six patients developed LBBB, followed by PR interval prolongation in four patients, and only two patients presented multiple conduction disturbances; 60% of the LBBB cases and all the cases of first- and second-degree atrio-ventricular block disappeared at 1 year post-TAVI. One patient required PPM for complete heart block, and several comorbidities were observed: previous LBBB and multiple prior cardiac surgeries, including a valve-in-valve procedure. No significant association was observed between the incidence of conduction disturbances after TAVI and risk factors among adult patients, such as right bundle branch block, prosthesis oversizing, valve implantation depth, and membranous septum length. After a median follow-up of 98 days, no newly diagnosed conduction disturbances, reintervention, and deaths were detected. Regarding the presence of rhythm disorders in pediatric patients following TAVI, no patients experienced severe or persistent arrythmias. The conduction system of pediatric patients is distinct, with faster conduction, shorter refractory periods, and less fibrosis than in adult patients, and the left ventricle outflow tract is more adaptable, which could explain the low and transitory incidence of conduction abnormalities [44].

The SwissTAVI registry, a prospective multicenter observational study, sought to investigate the 1-year all-cause mortality of PPM patients after TAVI, dividing the participants into three groups as follows: patients with a previous PPM; patients with need of a PPM during the first 30 days post-TAVI; and patients without a PPM. A total of 15% of the patients required PPM implantation during the first 30 days post-TAVI; these were patients who were at higher surgical risk (STS-PROM 4.7 ± 3.5 vs. 4.3 ± 3.7; *p* < 0.001), older (82 ± 6 years vs. 81 ± 7 years; *p* < 0.001), and more likely to present comorbidities—such as atrial fibrillation (34% vs. 29%), diabetes (29% vs. 25%; *p* < 0.001), or hypertension (82% vs. 80%, *p* = 0.003)—compared to patients who didn’t require a PPM. Self-expanding and mechanically expanding valves (MEV) were more frequently used among patients requiring a PPM than non-PPM patients (SEV 52% vs. 46%, *p* < 0.001; MEV 5.8% vs. 1.8%; *p* < 0.001). After multivariate adjustments, the following results were observed. Patients requiring a PPM had higher adjusted overall (aHR: 1.15; 95% CI 1.05–1.26; *p* = 0.002) and cardiovascular mortality (aHR: 1.25; 95% CI 1.06–1.46; *p* = 0.006) than non-PPM-requiring patients. Higher adjusted all-cause (aHR: 1.16; 95% CI 1.07–1.26; *p* < 0.001) and cardiovascular mortality (aHR: 1.18; 95% CI 1.04–1.36; *p* = 0.013) was found among these patients at 5 years, as well as at 10 years (all-cause mortality aHR: 1.16; 95% CI 1.07–1.25; *p* < 0.001 and cardiovascular mortality aHR: 1.18; 95% CI 1.04–1.34; *p* = 0.01), compared to among non-PPM patients. A reduction of ≥ 10% in the left ventricular ejection fraction was more frequent in patients receiving a PPM than in non-requiring patients (aHR: 1.88; 95% CI 1.72–2.05; *p* < 0.001), as well as in prior PPM patients (aHR: 1.37; 95% CI 1.11–1.69; *p* = 0.003), at 1 year. Compared to patients without a PPM, PPM-requiring patients presented higher rates of all-cause death, an LVEF decline of ≥10%, and NYHA class III-IV (aHR: 1.34; 95% CI 1.28–1.41; *p* < 0.001) [45].

PPM-receiving patients had different echocardiographic characteristics, such as the mean LVEF (56% ± 13% vs. 52% ± 14%; *p* < 0.001), mean transvalvular mean gradient (41 ± 16 mm Hg vs. 35 ± 16 mm Hg; *p* < 0.001), and valve area (0.77 ± 0.24 cm^2^ vs. 0.8 ± 0.3 cm^2^; *p* < 0.001), compared to prior PPM patients at baseline. In terms of overall mortality (aHR: 0.97; 95% CI 0.77–1.23; *p* = 0.82) and cardiovascular mortality (aHR: 0.9; 95% CI 0.63–1.29; *p* = 0.57), similar results were found compared to in prior PPM patients at one year post-TAVI. These outcomes persisted at 5- and 10-year follow-up, with similar overall (5-year aHR: 1.01, 95%CI 0.82–1.25; *p* = 0.907; 10-year aHR: 1.01, 95% CI 0.83–1.23; *p* = 0.924) and cardiovascular mortality (5-year aHR: 1.06, 95% CI 0.82–1.36; *p* = 0.671; 10-year aHR: 1.05, 95% CI 0.82–1.34; *p* = 0.716) between PPM-requiring and prior PPM patients. Higher composite rates in cardiovascular death, an LVEF decline of ≥ 10%, and NYHA III-IV were found among PPM-requiring patients and prior PPM patients (aHR: 1.2; 95% CI 1.05–1.38; *p* = 0.01) [45].

Even though the patients included in the SwissTAVI registry were not young, the results are important in regard to the long-term outcomes of PPM implantation. The higher overall mortality among PPM-requiring patients outlines the importance of other pacing methods for achieving a more physiological effect, such as conduction system pacing, which could be protective in the development of PICM. The higher composite rates in all-cause death, reduction in ejection fraction, and NYHA class among new PPM patients compared to prior PPM patients could be explained by the fact that conduction disturbances post-TAVI occur more suddenly, and also by the indication of the prior pacemaker, which could have preserved the atrio-ventricular conduction system [45].

Clinical practice: Even though not all PPM patients develop PICM, and it is important to recognize risk factors in advance in order to prevent complications. With LBBB being the most common conduction abnormality diagnosed after TAVI, and a previously described risk factor for the development of PICM, particular consideration should be given to patients with this condition, specifically those of younger age. Moreover, regular follow-up of patients who are at increased risk for PICM is recommended, as the time to diagnosis could be relative. The higher mortality observed in patients with permanent pacemakers is a warning sign to be taken into consideration, especially among younger patients, among whom high survival is desired.

Still, according to the presented information above, younger patients seem to have a lower risk of needing PPM implantation [41,42,44,45]; however, they could present comorbidities that increase the risk of needing PPMs [42,45]. Higher long-term mortality has been demonstrated in patients requiring a PPM after TAVI [45], but not yet in younger patients, and thus this represents a gap in young-patient data that should be addressed in further studies.

With the development of transcatheter aortic valve implantation techniques, the incidence of paravalvular leaks in patients treated with TAVI has begun to decrease; however, it has remained higher compared with patients treated with SAVR, a fact confirmed in the NOTION-2, PARTNER-3, and Evolut Low-Risk clinical trials [20,31,33,46]. Factors that increase the risk of developing PVL include calcium deposits in the left ventricular outflow tract, which influence the positioning of the prosthesis, as well as bicuspid aortic valves. Paravalvular leaks are associated with a worse post-procedural prognosis, including heart failure, infective endocarditis, hemolysis with debilitating symptoms, kidney injury, and life-threatening bleeding [46]. According to a study conducted by Okuno et al. [47], increased five-year all-cause mortality risk was observed in patients treated with TAVI who presented with mild-to-moderate post-procedural PVL compared to those with no/trace PVL (aHR, 1.56, 95% CI 1.20–2.02, *p* = 0.001). In such cases, additional treatment methods may be implemented, such as balloon postdilatation, Valve-in-Valve procedures, or paravalvular leak closure [47]. Thus, in young patients, higher rates of PVL could be expected, especially as bicuspid aortic valves have a higher incidence among these patients; however, early diagnosis and application of supplementary treatment methods when necessary is vital.

Stroke, although of low incidence among TAVI patients, is a dreaded complication due to its association with high mortality [40]. Although stroke in younger patients is associated with lower mortality compared with stroke in the elderly population, mortality in these patients remains higher compared with in the general population, and in addition, complications from cerebral events significantly reduce patients’ quality of life [48]. Most post-TAVI cerebral events are strokes, half of which are disabling, and the risk of their occurrence increases in high-risk patients. The main mechanism is dislodgement of plaques or calcifications caused by catheter and prosthesis manipulation at the aortic valve, while secondary mechanisms include vascular access, altered prosthetic hemodynamics post-intervention, the procedure itself, and patient comorbidities [46]. Another risk factor for stroke is the presence of extensive calcifications in the left ventricular outflow tract [49]. With the occurrence of embolic protection devices (Sentinel), their ability to capture emboli during the procedure has been demonstrated, although it remains inconclusive whether their use reduces the incidence of stroke [46]. Compared with that in SAVR patients, the risk of stroke in young TAVI patients is similar or lower, according to the DEDICATE, Evolut Low-Risk, and PARTNER-3 clinical trials; however, in the NOTION-2 clinical trial, the stroke risk in patients with bicuspid aortic valves was higher at one year compared with that in patients with bicuspid valves treated with SAVR [20,30,32,33]. Although less frequent, stroke may occur long after the procedure, and a possible explanation could be subclinical leaflet thrombosis, visualized on imaging techniques as hypoattenuated leaflet thickening, considered a prosthetic valve complication that leads to bioprosthetic valve dysfunction [49,50,51]. Along these lines, even though younger patients could present lower mortality after stroke than the elderly, prevention is key in order to maintain an increased quality of life.

Coronary obstruction, although rare, is associated with high mortality and may occur both during the intervention and post-procedurally. There are two main mechanisms for coronary obstruction: directly, during the implantation of the prosthesis—with the rearrangement of the calcific leaflets toward the coronary ostia; or indirectly, by leaflet expansion above the sinotubular junction, which causes sinus sequestration. The risk increases in patients with small sinuses and sinotubular junctions, extensive calcifications, and low-lying coronary ostia, and in Valve-in-Valve procedures [52].

With more younger patients undergoing TAVI, maintaining coronary access is vital, as their longer life expectancy could imply future coronary angiographies and interventions. The CAvEAT multicenter prospective registry studied the feasibility of coronary artery cannulation directly after TAVI with BEVs, as well as with SEVs. The BEV used was the intra-annular, short-frame SAPIEN3/Ultra; the SEVs used were Evolut Pro or Evolut Pro + (tall-frame, supra-annular, small-cell design), ACURATE neo/neo-2 (tall-frame, supra-annular, open-cell architecture), and Portico or Navitor (tall-frame, intra-annular, larger-cell design). A total of 632 patients, with a mean age of 82 years and a mean STS score of 3.2%, were divided equally to undergo TAVI with each valve type, resulting in 158 patients per valve. The primary endpoint considered was selective cannulation of the left and right coronary arteries. Coronary access was defined as selective if the catheter was in the coronary ostia, unselective if the coronary artery could be visualized but without the catheter in the coronary ostia, and unsuccessful in the case of non-visualization. A total of 49% of patients treated with BEVs presented coronary artery disease, as compared to 23% of patients treated with Evolut and Portico/Navitor, and 28% treated with ACURATE neo/neo2 prosthesis, respectively (*p* < 0.001). The highest rates of selective coronary artery access were obtained with the SAPIEN 3/Ultra valve (89%, 95% CI 84–93%), as compared to Portico or Navitor (63%, 95% CI 55–70%), ACURATE neo/2 (62%, 95% CI 53–69%), and Evolut Pro/+ (45%, 95% CI 37–53%), *p* < 0.001. Low rates of unfeasible cannulation were noticed, primarily involving the right coronary artery (5.5% vs. 0.5% for the left coronary artery), without statistically significant differences between the four prostheses (*p* = 0.06). Comparing the SEVs, the lowest rates of selective CA cannulation were observed with the Evolut Pro/+ heart valve. Unfeasibility or non-selective CA were more often experienced with a higher implantation depth (OR, 0.83, 95% CI 0.74–0.94, *p* = 0.002), in cases of moderate or severe misalignment (OR, 5.51, 95% CI 3.38–9.00, *p* < 0.001), and with the use of tall-frame valves (OR, 6.24, 95% CI 3.10–12.6, *p* < 0.001). When choosing a tall-frame heart valve, size was observed to be important only when implanting the Evolut Pro/+ valve, as higher rates of successful cannulation were obtained using larger sizes as compared to a smaller size (79% vs. 21%, *p* = 0.005). Moreover, there was one case of valve embolization during coronary cannulation after TAVI with the Evolut Pro valve [53].

At 30-day follow-up, secondary endpoints such as death, stroke, myocardial infarction, acute kidney injury, and PPM need were observed. Higher rates of permanent pacemaker need were found when using the Portico/Navitor (27%) or Evolut Pro/Pro+ (17%) valves as compared to SAPIEN 3/Ultra (7%) and ACURATE neo/2 (7%) (*p* < 0.001). Other than that, no significant differences were observed in the clinical outcomes. As such, tall-frame heart valves imply technical difficulties in obtaining coronary access after TAVI, which are most pronounced in small-cell designs (Evolut Pro/Pro+ valves) [53].

Ojeda et al. [54] led a prospective study observing the incidence, clinical presentation, management, in-hospital and 1 year mortality, and clinical outcomes of patients with coronary obstruction after TAVI—among 13.675 patients, 115 presented coronary obstruction. The clinical presentation of patients varied from acute coronary syndrome (46.1% of patients, more often non-ST-elevated myocardial infarction—52.8%) to cardiac arrest (41.7%), cardiogenic shock (7.8%), and unstable angina (4.3%). A total of 7.8% of the patients presented with obstruction of both the left and right coronary artery, and 68.7% with obstruction of the left coronary artery. Management of CO patients included percutaneous coronary intervention—PCI (89.6%), with a success rate of 78.0%; coronary artery bypass (3.5%); and no intervention (9.6%). Acute CO was more frequent with the use of BEVs (95.8% vs. 74.6%, *p* = 0.002), while delayed CO was more frequent with the use of SEVs (4.2% vs. 25.4%, *p* = 0.002). The PCI success rate was lower with SEVs than with BEVs (69.1% vs. 88.9%, *p* = 0.017). CO occurred more commonly among patients who underwent Valve-in-Valve procedures (21.7% vs. 3.1%, *p* = 0.001) [54].

Although the incidence of CO was low (0.80%), mortality rates were high. In-hospital mortality was higher among patients with CO compared to patients without (37.4% vs. 4.1%, *p* = 0.001), and varied with the timing of diagnosis, with a later diagnosis increasing the mortality risk (66.7% during hospitalization vs. 35.4% with diagnosis during the intervention, *p* = 0.046). Unsuccessfully treated or non-treated patients with CO presented higher mortality compared to treated CO patients (78.7% vs. 20.7%, *p* = 0.001) [54].

A total of 18.2% of patients with CO underwent coronary protection during the procedure, and lower mortality was observed in these patients (23.8% vs. 40.4%, *p* = 0.15). At 30 days, the mortality rate in patients with coronary protection was 28.6%, compared to 40.4% in patients without coronary protection (*p* = 0.31) respectively 28.6% vs. 41.5% (*p* = 0.273) at 1 year [54].

Within 30 days, mortality rates were higher among CO patients (38.3% vs. 4.3%, *p* < 0.001). At 1 year, overall mortality was 9.4%, with 39.1% among CO patients versus 9.1% in the control group (*p* < 0.001). After propensity score-matching, death (in-hospital or in the first 30 days) was observed in 38.3% of CO patients compared to 9.6% of non-CO patients, and the probability of mortality remained higher among CO patients at 1 year (39.1% vs. 13.9%, *p* < 0.001). Hence, although of low incidence, coronary obstruction carries a significant risk of death. According to Ojeda et al. [54], delayed coronary obstruction could be overlooked, as many patients receive conservative medication for acute coronary syndromes because of comorbidities and other procedural factors. Even though anatomical risk factors for CO are well known, this complication can also arise incidentally, and the PCI success rate in these cases is low [54].

Implantation depth is of utmost importance, as it could determine future coronary access, as well as induction of conduction disturbances requiring PPM, and thus, individualized implantation approaches should be considered [53].

This complication has contributed to the development of new-generation valves, such as the SAPIEN X4 and Evolut FX, which offer coronary access after TAVI and enhance commissural alignment visualization—techniques that are effective in preventing coronary artery obstruction (such as the Chimney technique, BASILICA)—as well as devices designed for this purpose, such as ShortCut, Splitter, and TELLTALE [55]. Furthermore, due to the possibility of progression of ischemic coronary heart disease, with the occurrence of acute coronary syndromes or coronary occlusion in young patients, attention must be paid to coronary access after TAVI, which is influenced by anatomical factors (sinus diameter, height and width of the sinotubular junction, and coronary artery location), as well as by procedural factors (prosthesis stent height, cusp position, and implantation technique) [56].

Clinical practice: As previously stated, patients with coronary obstruction present high mortality. Even though its incidence is low, identifying patients at increased risk of coronary artery disease and anatomical risk factors, as well as selecting the right prosthesis and performing additional techniques when necessary in each individual case, could help to prevent this complication. However, as coronary obstruction can appear incidentally, being wary of this complication is vital.

According to this information, SEVs are associated with lower success rates of PCI after coronary artery obstruction, selective coronary artery cannulation, and higher rates of PPM need—with the exception of ACURATE neo/2 [53,54]. Thus, BEVs could be the preferred approach in younger individuals undergoing TAVI, as coronary access and PPM need is of major interest among these patients.

## 7. Durability of Post-TAVI Valves and Reintervention Strategies

Surgically implanted mechanical prostheses are more durable; nevertheless, there is an upward trend among young patients regarding transcatheter aortic valve implantation, due to the possibility of avoiding open-heart surgery and lifelong anticoagulant therapy, while offering a faster recovery [57].

The durability of the transcatheter-implanted valve is one of the most important post-procedural concerns, especially among young patients, and it is well known that all biological prostheses degenerate over time [50].

Bioprosthetic valve dysfunction (BVD) is an entity consisting of four possible complications of the prosthesis: structural valve deterioration (SVD), non-structural valve dysfunction (NSVD), thrombosis, and prosthetic valve endocarditis. By contrast, bioprosthetic valve failure (BVF) is defined as prosthesis-related death (as a result of structural valve deterioration), severe hemodynamic structural deterioration, and the need for reintervention due to the diagnosis of SVD [38,50,51] (Table 5).

Severe structural deterioration is defined as a transprosthetic gradient of ≥30 mmHg, an increase of ≥20 mmHg within the last 3 months associated with a decrease in the effective orifice area of ≥0.6 cm^2^ or in Doppler velocity index ≥0.2 or ≥40%, or newly documented severe intraprosthetic regurgitation. This occurs due to procedure-related parameters such as the type of biological valve and the use of undersized prostheses, which increase the transprosthetic gradient. Clinically, young patients, smokers, and those with a high body mass index, dyslipidemia, diabetes, or chronic kidney disease are predisposed to structural valve deterioration. Anatomical parameters could also predispose an individual to reduced prosthesis durability, for example a small aortic annulus diameter or bicuspid aortic valves [38,50,51].

Post-TAVI, the presence of atrial fibrillation is associated with SVD, which could predispose patients to leaflet thrombosis [59].

In the diagnosis of SVD, the following steps are recommended [59]:

- Assessing clinical symptoms or new echocardiographic changes;
- Identifying the etiology of SVD;
- Severity staging;
- Analyzing the outcomes of BVF.

Long-term post-interventional results have now become available. The NOTION clinical trial compared the results of TAVI with SEVs with SAVR in 280 patients (enrolled between 2009 and 2013) in regard to clinical and durability outcomes. Patients aged 70 or older with symptomatic severe degenerative AS or asymptomatic AS with left ventricular (LV) dysfunction (<60%, but >20%), hypertrophy (LV wall thickness ≥ 17 mm), or atrial fibrillation, who were eligible for both interventions and had a life expectancy of ≥ 1 year, were included. Exclusion criteria encompassed hemodynamic instability, certain severe comorbidities, and other valve-related parameters, such as isolated aortic valve insufficiency. Primary outcomes were composed of all-cause death, stroke, and myocardial infarction within the first year, while exploratory outcomes included clinical and echocardiographic data relating to valve performance [51,60].

In the NOTION trial, the mean age of the participants was 79.1 ± 4.8 years, with a mean STS-PROM score of 3.0 ± 1.7%. After 10 years, 36.1% of patients were alive. Clinically, no difference in all-cause mortality was observed (62.7% for TAVI vs. 64.0% SAVR, HR 1.0, 95% CI 0.7–1.3, *p* = 0.8), as well as in all-cause mortality, stroke, or MI (65.5% TAVI vs. 65.5% SAVR, HR 1.0, 95% CI 0.7–1.3, *p* = 0.9). Regarding newly diagnosed atrial fibrillation, a higher incidence was observed in SAVR patients than in TAVR patients at any time of follow-up (74.1% vs. 52.0%, *p* < 0.01). Rates of permanent pacemaker implantation were higher in the TAVI cohort (44.7% vs. 14.0%, *p* < 0.01) [51].

Echocardiographic outcomes showed a decrease in the mean transprosthetic gradient with an increase in the effective orifice area initially, with the gradients and area more favorable in the TAVI cohort (*p* < 0.05); however, over time, the gradients increased and EOA decreased in both groups. Among TAVI-treated patients, more moderate-to-severe PVL and bioprosthesis regurgitation was observed after 10 years, and at any point during the 10 years, TAVI patients had a higher risk of moderate-to-severe PVL (25.4% vs. 2.5%, *p* < 0.01). Paravalvular leak at 3 months post-procedure did not affect all-cause mortality at 10 years (moderate/severe PVL 62.0% vs. no/mild PVL 55.5%, *p* = 0.84) [51].

The risk of moderate or severe SVD was similar between the two interventions (15.4% after TAVI vs. 20.8% after SAVR, HR 0.7, 95% CI 0.4–1.2, *p* = 0.3); however, TAVI patients had a lower risk of severe structural valve deterioration (10.0% vs. 1.5%, HR 0.2, 95% CI 0.04–0.7, *p* = 0.02), as well as of severe bioprosthetic valve dysfunction (43.0% vs. 20.5%, *p* < 0.01) and non-structural valve deterioration (31.9% vs. 10.2%, *p* < 0.01). Clinical valve thrombosis was not observed in any patient, and low rates of infective endocarditis were identified in both groups (7.2% TAVI vs. 7.4% SAVR, *p* = 1.0). No differences in bioprosthetic valve failure were seen between patients (9.7% in TAVI vs. 13.8% in SAVR, HR, 0.7, 95% CI 0.4–1.5, *p* = 0.4). The rates of reintervention were low among both groups (4.3% in TAVI vs. 2.2% in SAVR, *p* = 0.3), mostly for restenosis (five patients in TAVI vs. two in SAVR) and central regurgitation (one each), and for all reinterventions, TAVI was performed [51].

According to this clinical trial, transcatheter-implanted valves did not show early degeneration, but neither did they show superior durability compared with surgically implanted valves. It is important to note that first-generation transcatheter valves were used, while some surgically implanted valves are known for having lower durability, and more SAVR patients exited the trial. To date, these are the only available data regarding long-term follow-up of TAVI-treated patients [50,51].

The results of the PARTNER-3 clinical trial revealed similar durability of transcatheter and surgically implanted prostheses at 5 years post-intervention. The mean transprosthetic gradients (12.8 ± 6.5 mmHg vs. 11.7 ± 5.6 mmHg), as well as the aortic valve area (1.9 ± 0.5 cm^2^ vs. 1.8 ± 0.5 cm^2^), were similar between TAVI and SAVR patients. Bioprosthetic valve failure of any cause was estimated at 3.3% for TAVI vs. 3.8% for SAVR (HR, 0.86. 95% CI 0.42–1.77), and severe structural valve deterioration was estimated at 1.1% for TAVI and 1.00% for SAVR, with bioprosthetic valve failure related to SVD at 1.4% vs. 2.0%. After 5 years, 86.3% of TAVI patients and 87.4% of SAVR patients were alive with normally functioning valves. Reintervention rates were similar between the two groups (2.6% for TAVI vs. 3.0% for SAVR, HR, 0.86, 95% CI 0.39–1.92) [32].

The durability of transcatheter heart valves at mid-term after the intervention, with the exception of first-generation BEVs, is similar to that of SAVR valves [59].

Several options exist in the treatment of bioprosthetic valve failure. However, prevention is also important, which includes anticipating the risk of certain complications. Patient–prosthesis mismatch, which could lead to NSVD, is more frequent after SAVR, and patients with a small aortic annulus are at increased risk; thus, TAVI may be an optimal choice among these patients. Performing balloon predilatation or postdilatation could also prevent severe patient–prosthesis mismatch, but at the cost of other complications, such as aortic annulus rupture or paravalvular regurgitation. Considering the hypothesis that structural valve deterioration occurs because of fibrocalcific degeneration caused by mechanical stress, in conjunction with bioprosthetic antigens that could trigger an immune response and pre-treatment of bioprosthesis with glutaraldehyde, treating patients with anti-inflammatory medication and lipid-lowering drugs could help with prevention [59].

There are multiple interventions that could be performed in cases of bioprosthetic valve failure. TAVI explant consists of the surgical removal of the transcatheter biological prosthesis, a procedure associated with increased mortality and a higher risk of stroke, while redo-SAVR refers to the surgical replacement of the dysfunctional prosthesis [40].

Redo-TAVI is further divided into two categories depending on the primary intervention: Valve-in-Valve TAVI (ViV TAVI), where the initial prosthesis is surgically implanted, and TAVI-in-TAVI, where the initial prosthesis is a transcatheter [40]. Redo-TAVI increases the risk of coronary obstruction, as the first valve leaflets expand and create a neoskirt that could adjoin the coronary ostia or isolate the sinotubular junction. Even though there are various neoskirt lengths, the implantation depth is also important [61].

Tang et al. [62] presented the mid-term outcomes of 396 patients who underwent either TAVI-explant or redo-TAVI for transcatheter valve failure from the EXPLANTTOREDO-TAVR International Registry. Patients with the second intervention performed during the index hospitalization, emergency surgery patients, and endocarditis patients were excluded. The interventions were performed for SVD (58.2%), more-than-moderate PVL (30.9%), severe patient–prosthesis mismatch (8.3%), valve thrombosis (2.9%), and delayed prosthetic valve migration. A total of 54.3% of patients underwent redo-TAVI, while 45.7% underwent TAVI-explant. Primary outcomes encompassed cumulative, in-hospital, 30-day, and 1-year mortality. The median interval to reintervention, median hospital length of stay, in-hospital complication rates—such as stroke, vascular complications, PPM implant, and life-threatening bleeding—and 30-day stroke rates were observed as secondary outcomes. Reintervention was labeled as emergency, urgent, or elective, considering the timing (<6 h after diagnosis of valve failure, during the hospitalization, or on a separate admission) [62].

Patients who underwent redo-TAVI were older compared to TAVI-explant patients (78.6 ± 8.4 years vs. 72.1 ± 9.0 years, *p* < 0.001), and had a higher heart team-determined surgical risk (high/extreme risk 63.4% vs. 37.5%, *p* < 0.001) and a more calcified aorta (24.5% vs. 6.4%, *p* < 0.001). TAVI-explant patients had more previous PCI (29.6% vs. 5.4%, *p* < 0.001) and cardiac surgery (49.2% vs. 27.2%, *p* < 0.001), and the median interval from index TAVI was shorter (17.6 months, IQR: 5.0–40.7 months) among these patients compared to among redo-TAVI patients (45.7 months, IQR: 10.6–75.6 months), *p* < 0.001 [62].

Considering the mechanism of valve failure, SVD was treated by redo-TAVI more frequently (63.7% vs. 51.9%, *p* = 0.023), while TAVI-explant was more frequently carried out for severe patient–prosthesis mismatch (17.1% vs. 0.5%, *p* < 0.001) and delayed valve migration (3.3% vs. 0.5%, *p* = 0.055). However, no difference among the indication for reintervention for valve thrombosis (3.9% redo TAVI vs. 1.7% TAVI-explant, *p* = 0.23) and PVL (32.8% redo-TAVI vs. 28.7% TAVI-explant, *p* = 0.44) was observed [62].

A total of 29.3% of reinterventions were urgent or emergency, more often in the TAVI-explant group (38.6–20.8%, *p* < 0.001). Those in the TAVI-explant group who also underwent aortic root replacement (10.5% of patients) received a bioprosthesis in 89.5% of cases. A TAVI-explant without aortic root replacement was implanted with a bioprosthesis in 85.8% of cases, while the rest received mechanical valves. In this patient cohort, additional procedures were performed in 55.8% of cases during reintervention, such as mitral valve surgery (20.4%), tricuspid valve surgery (2.8%), coronary artery bypass grafting (17.7%), ascending aortic replacement (6.1%), and aortic root repair (1.7%) [62].

Considering the type of the first transcatheter valve, no differences in regard to reintervention strategy were observed, with those with BEV failure undergoing redo-TAVI in 54.7% of cases and TAVI-explant in 45.3% (*p* = 0.92), while 54.0% of those with non-BEV failure underwent redo-TAVI and 46.0% underwent TAVI-explant (*p* = 0.92). Non-BEVs were more frequently used with BEV failure (58.6% vs. 41.4%, *p* = 0.036), while non-BEV failure was mostly treated with BEVs (56.3% vs. 43.8%, *p* = 0.038) [62].

Higher rates of in-hospital mortality were observed in TAVI-explant patients (11.6% vs. 2.8%, *p* = 0.001), who also had longer hospitalization stays (11 days, IQR: 7–17 days vs. 5 days, IQR: 2–7 days, *p* < 0.001). The redo-TAVI patients presented more vascular complications (12.3% vs. 2.9%, *p* = 0.001), 1.9% of cases were converted to open surgery, and one case of coronary obstruction was identified. Echocardiographic outcomes showed similar transvalvular gradients (12.2 ± 6.7 mmHg after redo-TAVI vs. 11.8 ± 5.7 mmHg, *p* = 0.67); however, a greater rate of more-than-moderate residual PVL was observed after redo-TAVI (5.6% vs. 0%, *p* < 0.001) [62].

The mortality rate at 30 days was 8.0%, and was higher among TAVI-explant patients (13.6% vs. 3.4%, *p* < 0.001). At 30 days, stroke and readmission rates of 3.4% and 13.8%, respectively, were observed. At one year, mortality was 22.3%, and was significantly higher in the TAVI-explant cohort (32.4% vs. 15.4%, *p* < 0.001), and the stroke rate was 5.3%. No significant difference was observed between the interventions regarding stroke rates both at 30 days (4.2% after redo-TAVI vs. 2.4%, *p* = 0.40) and at 1 year (5.8% after redo-TAVI vs. 4.6%, *p* = 0.78). Following landmark analysis, after 30 days, similar rates of mortality were observed (*p* = 0.91). Independent predictors of mortality found after multivariable Cox regression after redo-TAVI were chronic kidney disease (HR 4.11, 95% CI 1.85–9.15), the risk determined by the heart team (HR 2.16, 95% CI 1.24–3.77), and urgent/emergency redo-TAVI (HR 3.21, 95% CI 1.35–7.65). TAVI-explant independent mortality predictors were dialysis (HR 3.30, 95% CI 1.42–7.68), pulmonary hypertension (HR 2.34, 95% CI 1.22–4.50), and concomitant mitral surgery (HR 2.34, 95% CI 1.17–4.66) [62].

According to the EXPLANTTOREDO-TAVR registry, the incidence of reintervention following valve failure was low (0.59%), and SVD was mostly treated with redo-TAVI, while patient–prosthesis mismatch was more frequently treated with TAVI-explant. Higher in-hospital, 30-day, and 1-year mortality was observed in the TAVI-explant cohort, although after landmark analysis, no significant differences were observed regarding mortality after 30 days (4-year mortality of approximately 30%). Both interventions presented independent risk factors for increased mortality: in the redo-TAVI group, the factors were chronic kidney disease, a high risk at reintervention, and the emergency of the procedure, while for the TAVI-explant group, the factors were dialysis, pulmonary hypertension, and concomitant mitral surgery. Given the latter risk factor, in patients with multivalvular disease (i.e., concomitant aortic and mitral valve), surgery could be preferred as the standard treatment, as TAVI with deep implantation could lead to secondary mitral valve dysfunction [62].

**Figure 1:**
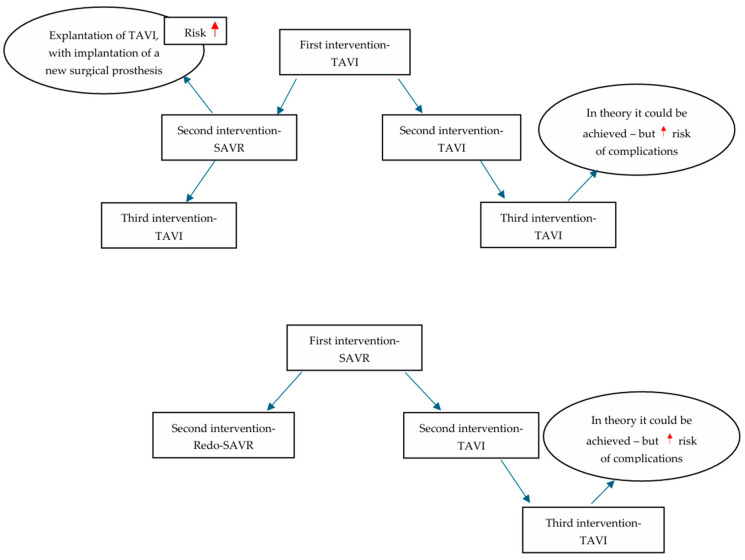
Possibilities of reintervention depending on the primary intervention, modified from [64]. TAVI = transcatheter aortic valve implantation; SAVR = surgical aortic valve replacement.

It is expected that the survival of young patients undergoing TAVI or SAVR with biological valves, especially those at low surgical risk, will exceed the durability of the valve. Thus, the initial management of these patients is very important, since future reinterventions will be guided by it [63]. In theory, multiple reintervention options exist; however, their feasibility is dictated by patients’ anatomical characteristics, the type of first implanted prosthesis, and the implantation technique, as well as the cause of valve failure and, ultimately, patient preferences [61,63]. Even though repeat TAVI is less invasive, it additionally predisposes individuals to the aforementioned risks [63]. As TAVI is now more frequently performed in younger patients, the potential strategy of reintervention with ultimately three prostheses—TAVI -TAVI -TAVI—raises questions, as the risks of the aforementioned complications increase considerably. Still, the gold standard so far among younger patients remains SAVR-SAVR-TAVI or SAVR-TAVI-TAVI, at least until further evidence is obtained. However, the role of the heart team in decision-making remains pivotal [64] (Figure 1).

Clinical practice: The findings of the NOTION trial are encouraging in terms of valve durability, deterioration, and reintervention rates among TAVI patients. Even though the patients included were older, they showed favorable long-term outcomes in regard to valve performance, although with limitations, as mentioned. The PARTNER-3 trial suggests similar results, with low rates of structural valve deterioration, bioprosthetic valve failure, and reintervention; however, these are limited to 5 years. Considering reintervention strategies, short-term mortality favors redo-TAVI; however, after landmark analysis, the longer-term mortality risk was found to be similar. In regard to young patients, as SVD could be more prevalent in these patients, and there is a higher incidence of bicuspid aortic valves among them, increased rates of reintervention could be expected; however, long-term results of major trials are awaited for a more comprehensive understanding. Thus, we underline the importance of initial management, with an ultimate goal of increased durability, low rates of complications, and lifetime management.

Currently, there are limitations regarding younger patients in terms of valve durability, as long-term results are not yet available. However, according to Yamamoto et al. [65], better valve performance and lower rates of PVL and patient–prosthesis mismatch were observed with the use of SAPIEN 3 Ultra RESILIA than with the SAPIEN 3 valve. This new-generation valve contains anticalcification technology [65], and could provide an improvement in terms of durability, especially among younger patients; however, more research is needed.

## 8. Conclusions

Aortic stenosis, the most frequent pathology requiring valve replacement interventions, is associated with high mortality in the absence of appropriate treatment, and in young patients, the most common etiologies are bicuspid aortic valves and rheumatic heart disease.

Associated anatomical particularities of BAVs, such as high degrees of calcification or aortopathy, contribute to higher post-TAVI mortality rates. The main limitation of TAVI in BAV patients is the higher incidence of complications and mortality, a fact confirmed by the NOTION-2 trial; however, the importance of bicuspid aortic valve morphology characterization is outlined in the study conducted by Li et al. [21], with lower all-cause and cardiovascular long-term mortality in type 0 BAVs.

In acute rheumatic fever patients, TAVI outcomes appear to be similar to those in patients with severe calcific aortic stenosis. However, different morphological changes involving the aortic valve, which could require different approaches during the intervention, and the absence of long-term data involving these patients could be limitations for TAVI.

Outcomes of TAVI among young patients are encouraging; however, it is very important to note that surgical risk is not always equivalent to patient age, with studies confirming higher post-TAVI mortality in young patients with multiple comorbidities. Young patients could present with congenital heart disease complications, with increased morbidity observed after TAVI, and in such cases, SAVR could be the preferred treatment method.

The presence of extensive calcifications in the left ventricular outflow tract could be a predictive factor for the occurrence of conduction disturbances, paravalvular leaks, and post-procedural strokes. Severe conduction disturbances could lead to permanent pacemaker implantation, which may predispose individuals to pacemaker-induced cardiomyopathy. Coronary obstruction, although rare, is a dreaded complication, and maintaining coronary access in young patients is essential due to the potential progression of ischemic coronary heart disease. Coronary obstruction and PICM are important factors to be taken into consideration when performing TAVI in young patients, as increased mortality is to be expected with these complications.

The durability of biological prostheses is a subject of great interest among young patients, as their survival may exceed that of the prosthesis, and it is already known that all biological prostheses degenerate over time. Moreover, young patients and bicuspid aortic valves present an increased risk of lower valve durability. Multiple options exist for the treatment of bioprosthetic valve failure, but their feasibility depends on the patient’s anatomical characteristics, the type of the first implanted prosthesis, and the implantation technique.

Until further studies are conducted, SAVR remains preferable in patients with AS secondary to BAVs, especially those that also present excessive calcifications or ascending aortic ectasia. SAVR may also be adopted in patients with ARF who present multivalvular involvement in cases that require double-valve replacement. Among younger patients, those with few comorbidities and patients with type 0 BAVs could represent subgroups where TAVI would be most beneficial, according to the information presented. However, more research is needed.

Thus, young patients treated with TAVI present survival outcomes similar to those treated with SAVR in the short-to-medium term after the intervention, though with certain possible complications, as mentioned. The continuous development of transcatheter valve technologies and future long-term results of the clinical trials cited will contribute to a clearer understanding of the implications of TAVI in young patients, with the aim of offering them optimal treatment, minimal complications, and increased valve durability. However, more research regarding durability, reinterventions, coronary access, and the implications of TAVI among BAV and ARF patients is essential.

## Figures and Tables

**Table 1:** Morphological types of bicuspid aortic valves, adapted from Hamana et al. [12] based on Sievers et al.

	Bicuspid Aortic Valve Type 0	Type 1	Type 2
Cusps	Pure bicuspid	2 cusps, unequal	2 cusps
Raphes	None	Raphe at the level of the fused cusp	2 raphes

**Table 2:** Division of patients into 3 groups according to pathology and intervention, adapted from Mentias et al. [23]. SAVR = surgical aortic valve replacement; TAVI = transcatheter aortic valve implantation.

Severe Rheumatic Aortic Stenosis	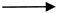	SAVR
Severe Rheumatic Aortic Stenosis	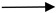	TAVI
Severe Non-Rheumatic Aortic Stenosis	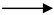	TAVI

**Table 3:** Outcomes in terms of mortality of the main presented studies, adapted from [20,25,26,27,28,29,30,31,32,33,34]. BEV = balloon-expandable valve; SEV = self-expanding valve; MEV = mechanically expandable valve; TAVI = transcatheter aortic valve implantation; SAVR = surgical aortic valve replacement; CHSD = Congenital Heart Surgery Database; ACSD = Adult Cardiac Surgery Database; RCT = randomized controlled trial.

	Study	Population	Age	Valve Type	Follow-Up	Primary Endpoints
Ancona et al. [25].	Single-center retrospective	129 high-risk-patients	Mean age 63.6 ± 6 years	1st- and 2nd-generation, use of BEV, SEV, MEV	3 years	34% all-cause mortality
Bække et al. [26].	Retrospective multicenter	459 TAVI-treated patients compared to 1836 matched patients	Mean age 61 years	-	3 years	34% mortality in TAVI group, especially in patients with prior comorbidities
Gad et al. [27].	Retrospective	3672 TAVI patients compared to matched SAVR patients	18–59	-	In-hospital	Similar in-hospital mortality to SAVR
Nelson et al. [28].	Retrospective	1580 records from CHSD and ACSD	18–55	-	30 days	Lower, but not statistically different, mortality after SAVR than TAVI
DEDICATE Trial	Prospective, multicenter, RCT	1414 symptomatic severe aortic stenosis patients at low–moderate surgical risk eligible both for SAVR and TAVI	Mean age 74 ± 4	BEV in 61.4% of TAVI patients	1 year	TAVI non-inferior regarding mortality and stroke
NOTION-2	RCT	370 severe AS patients at low surgical risk, including those with BAVs	Mean age 71.1	SEV in 72.7% of TAVI patients	1 year	Similarity to SAVR regarding all-cause mortality, stroke, rehospitalization
PARTNER-3	Multicenter RCT	950 low-surgical-risk patients, eligible for both TAVI and SAVR, with no major particularities	Mean age 73	BEV	1 year	TAVI superior in regard to risk of death, stroke, rehospitalization
PARTNER-3					5 years	TAVI similar to SAVR in terms of death, stroke, rehospitalization
Evolut Low Risk Trial	Multicenter, prospective, RCT	1414 Low surgical risk patients	Mean age 74	SEV	5 years	TAVI similar to SAVR regarding all-cause mortality or disabling stroke

**Table 4:** Incidence of complications in the main RCTs cited, adapted from [20,29,30,31,32,33,34]. TAVI = transcatheter aortic valve implantation; PPM = permanent pacemaker; PVL = paravalvular leak.

Trial	Stroke	PPM	Atrial Fibrillation	Valvular Outcomes	Reintervention
DEDICATE	Lower post-TAVI	Higher post-TAVI	Lower post-TAVI	Higher prosthetic valvular dysfunction post-TAVI	Apparently higher post-TAVI
NOTION-2	Higher post-TAVI	Higher post-TAVI	Lower post-TAVI	Higher rates of more-than-moderate PVL post-TAVI	Similar rates of reintervention
PARTNER-3	Lower post-TAVI after 1 year; similar after 5 years	Similar rates	Lower post-TAVI	Higher rates of PVL	Similar rates of reintervention
Evolut Low Risk	Similar rates	Higher post-TAVI	Lower post-TAVI	More-than-mild PVL higher post-TAVI	Similar rates of reintervention

**Table 5:** Definitions of the complications of bioprosthetic valve dysfunction, adapted from [38,50,51,58,59]. SVD = structural valve deterioration; NSVD = non-structural valve dysfunction.

Complication	SVD	NSVD	Thrombosis	Endocarditis
Definition	Direct damage to components through calcifications or fibrosis, or traumatic processes. Develops progressively.	Any impairment of the prosthesis not directly involving it—patient–prosthesis mismatch, paravalvular regurgitation. Usually occurs during the procedure and is permanent.	Clinically manifested thromboembolic event, or worsening of aortic stenosis/regurgitation, with demonstration of subclinical leaflet thrombosis. Could be reversible with medication, but also persistent and damaging.	Endocarditis diagnosed according to modified Duke criteria. Could be reversible with medication, but also persistent and damaging.

## Data Availability

No new data were created.
